# Predictive factors of adrenal insufficiency in patients admitted to acute medical wards: a case control study

**DOI:** 10.1186/1472-6823-13-3

**Published:** 2013-01-26

**Authors:** Jean-Baptiste Oboni, Pedro Marques-Vidal, François Pralong, Gérard Waeber

**Affiliations:** 1 Department of Medicine, Internal Medicine Unit, CHUV and Faculty of Biology and Medicine, Lausanne, Switzerland; 2 Institute of Social and Preventive Medicine (IUMSP), University Hospital of Lausanne, Lausanne, Switzerland; 3 Department of Medicine, Endocrine and Diabetes Unit, CHUV and Faculty of Biology and Medicine, Lausanne, Switzerland; 4 Department of Internal Medicine, BH-10-628, University Hospital CHUV, Lausanne, Switzerland

**Keywords:** Adrenal insufficiency, Prevalence, Risk factors, ACTH-stimulation test, Case-control study

## Abstract

**Background:**

Adrenal insufficiency is a rare and potentially lethal disease if untreated. Several clinical signs and biological markers are associated with glucocorticoid failure but the importance of these factors for diagnosing adrenal insufficiency is not known. In this study, we aimed to assess the prevalence of and the factors associated with adrenal insufficiency among patients admitted to an acute internal medicine ward.

**Methods:**

Retrospective, case-control study including all patients with high-dose (250 μg) ACTH-stimulation tests for suspected adrenal insufficiency performed between 2008 and 2010 in an acute internal medicine ward (n = 281). Cortisol values <550 nmol/l upon ACTH-stimulation test were considered diagnostic for adrenal insufficiency. Area under the ROC curve (AROC), sensitivity, specificity, negative and positive predictive values for adrenal insufficiency were assessed for thirteen symptoms, signs and biological variables.

**Results:**

32 patients (11.4%) presented adrenal insufficiency; the others served as controls. Among all clinical and biological parameters studied, history of glucocorticoid withdrawal was the only independent factor significantly associated with patients with adrenal insufficiency (Odds Ratio: 6.71, 95% CI: 3.08 –14.62). Using a logistic regression, a model with four significant and independent variable was obtained, regrouping history of glucocorticoid withdrawal (OR 7.38, 95% CI [3.18 ; 17.11], *p-value* <0.001), nausea (OR 3.37, 95% CI [1.03 ; 11.00], *p-value* 0.044), eosinophilia (OR 17.6, 95% CI [1.02; 302.3], *p-value* 0.048) and hyperkalemia (OR 2.41, 95% CI [0.87; 6.69], *p-value* 0.092). The AROC (95% CI) was 0.75 (0.70; 0.80) for this model, with 6.3 (0.8 – 20.8) for sensitivity and 99.2 (97.1 – 99.9) for specificity.

**Conclusions:**

11.4% of patients with suspected adrenal insufficient admitted to acute medical ward actually do present with adrenal insufficiency, defined by an abnormal response to high-dose (250 μg) ACTH-stimulation test. A history of glucocorticoid withdrawal was the strongest factor predicting the potential adrenal failure. The combination of a history of glucocorticoid withdrawal, nausea, eosinophilia and hyperkaliemia might be of interest to suspect adrenal insufficiency.

## Background

Adrenal insufficiency is a relatively rare but potentially lethal disease if missed during acute settings [1,2]. Symptoms, signs and biological markers associated with adrenal failure are well known by clinicians [3,4]. However the relative importance of these symptoms, signs and markers has not been fully studied in patients admitted in acute medical wards. Adrenal insufficiency is defined as primary or secondary. Autoimmune and tuberculous adrenalitis are the principal etiologies for primary adrenal failure, which is characterized by low cortisol levels and elevated plasma concentrations of ACTH. Impairment of the hypothalamic-pituitary corticotropic axis is responsible for secondary causes of adrenal insufficiency. These situations are characterized by low circulating levels of cortisol and ACTH. The most frequent cause of secondary adrenal insufficiency is a tumour of the hypothalamic-pituitary region but administration of supraphysiologic doses of glucocorticoids may alter a normal hypothalamic response with secondary adrenal failure once individuals are weaned from the glucocorticoid treatment. Bilateral adrenalectomy or drug-induced adrenal insufficiency may be considered as iatrogenic etiologies for adrenal failure.

Symptoms commonly associated with adrenal insufficiency are “fatigue” (lack of energy or stamina), abdominal pain, nausea, and dizziness (hypotension symptoms). The patient history may include a key element related to previous glucocorticoid treatment, thus increasing the risk of secondary adrenal failure related to glucocorticoid withdrawal. Signs associated with adrenal insufficiency are low blood pressure, vitiligo and/or skin changes. Biological markers of the disease include hyperkalemia, hyponatremia, acidosis, hypercalcaemia and eosinophilia. Nevertheless, the proportion of individuals with one or more symptoms or signs associated with adrenal insufficiency is not clearly described in the literature.

Evaluating adrenal function is a difficult task in clinical practice [5]. Cortisol response to ACTH administration may depend upon the biological stress of the patients (intensive care versus stable conditions), the cut-off values for cortisol levels and the use of low (1 μg) versus high (250 μg) dose ACTH-stimulation test [6-9]. Basal cortisol levels inaccurately assess adrenal function [10]. Conversely, the high dose ACTH-stimulation test (“Synacthen® test”, cosyntropin stimulation test or tetracosactide test) is generally accepted as reliable to evaluate adrenal function in everyday practice, and considered as the easiest to perform [11-13]. If this test is recognized as gold standard in various clinical situations, it may be more difficult to define cut-off values for cortisol levels in intensive care. Patel and co-authors described expected values of basal serum cortisol (> 250 nmol/l) and peak cortisol (> 600 nmol/l) after 250 μg intramuscular tetracosactrin in acute hospital admissions [14]. However, the optimal cut-off for peak cortisol levels avec ACTH challenge has been questioned by several authors and current recommendations seem to include a minimum of > 18-20 μg/dl (> 500–550 nmol/l) to consider the ACTH response as adequate. These normal values vary dependent on laboratory and assay. In our department, the 550 nmol/l cut-off is considered as a normal response to the ACTH challenge. The minimum increment in serum cortisol is considered by Patel [14] and others as invalid to diagnose adrenal failure, because individuals who have a high basal concentration, due to normal circadian rhytmicity or acute stress, may be unable to increase further cortisol secretion.

The aim of this study was to identify the clinical and biological determinants associated with adrenal insufficiency in patients admitted to an acute medical ward.

## Methods

### Study population

All patients admitted in a general internal ward of the Lausanne University Hospital between 1st of January 2008 and 31^st^ of December 2010 and requiring an ACTH-stimulation test based upon clinical judgment were included. This study was approved by the Institutional Ethics Committee of the University of Lausanne.

### Data collection

All patient files were reviewed, and information related to a potential adrenal insufficiency as well as reasons to order the ACTH-stimulation test were included in the analysis. Symptoms collected were: fatigue, abdominal pain, nausea, generalized weakness, hypotension symptoms such as diziness and/or history of glucocorticoid withdrawal. Physical signs collected were: low blood pressure (<100 mmHg systolic and/or <50 mmHg diastolic blood pressure), vitiligo and/or skin changes. Biological markers collected were: hyperkalemia (> 5 mmol/l), hyponatremia (< 135 mmol/l), acidosis (pH < 7.35), hypercalcaemia (total and ionized, corrected calcium  > 2.5 mmol/l) and eosinophilia (> 0.5 G/l). Except for age, all other variables were coded as yes/no. ACTH-stimulation test was considered as normal if any value of cortisol was  ≥ 550 nmol/l.

### Statistical analysis

Statistical analysis was conducted using R (http://www.r-project.org/) and Stata version 12 (Stata corp, College Station, TX, USA). Results were expressed as number of patients and (percentage) for qualitative data and as mean ± standard deviation for quantitative data. Chi -square analysis was used to compare categorical data between patients with abnormal ACTH-stimulation test and control group. A multivariate logistic regression analysis was performed to determine which variables were independently associated with an abnormal ACTH-stimulation test. Stepwise forward regression was used for model selection. Two models were used: a “restricted” model, with a significance level for entry < 0.05, and a “broad” model, with a significance level for entry <0.10. Results were expressed as Odds ratio (OR) and 95% confidence interval (CI). The performance of each model in discriminating between patients and controls was assessed using the area under the Receiver Operating Characteristic curve (AROC), the sensitivity, the specificity, the positive and the negative predictive values, with corresponding 95% CIs. Statistical significance was established with *P* less than 0.05.

## Results

### Patient selection

During the 36 months of the survey, 11’310 patients were hospitalized in the general internal ward. Among them, 339 (2.9%) were clinically suspected of adrenal insufficiency and had an ACTH-stimulation test. Unexplained signs such as hypotension, hyperkalemia, metabolic acidosis or hyponatremia were the key signs used by clinicians to order the ACTH-stimulation test in most clinical situations. If adrenal failure was suspected, the ACTH-test was performed and in most cases, stress doses of glucocorticoid administrated for 48 hours. If the short ACTH stimulation test was normal, the glucocorticoid treatment was immediately stopped. In the proven 32 cases of adrenal failure, stress doses of hydrocortisone or prednisone were administrated and tapered. All patients with adrenal insufficiency left the hospital under glucocorticoid treatment. Among these 339 patients, 6 (1.8%) were considered as false-positives as they were under glucocorticoid treatment and 52 (15.3%) were excluded because of missing information for at least one study variable. The ACTH-stimulation test was normal in these 52 individuals. The remaining 281 (82.9%) patients (149 women and 132 men) were retained for analysis.

### Patient characteristics

Among the 281 patients, 32 (11.4%) were abnormal responders (blunted response to the ACTH-stimulation test) while the remaining 249 (88.6%) were normal responders and served as controls. The clinical and biological characteristics of the patients with adrenal insufficiency and the controls are summarized in Table 1. For the 32 patients that had a confirmed adrenal failure, ACTH levels were available for 8 patients. On those 8 patients, 6 (18.7%) combined low levels of cortisol and elevated ACTH levels confirming the diagnostic of primary adrenal failure. The other 2 combined low levels of cortisol and ACTH, confirming a secondary adrenal failure. The final suspected diagnosis was secondary adrenal insufficiency for 26 patients (another 24 patients) (81.3%). Profound adrenal failure was identified for 14 patients (43.8% of all cases) with cortisol levels < 100 nmol/l. We cannot rule out that several suspected cases of secondary adrenal insufficiency have been inadequately classified as secondary since the ACTH levels were missing. However, a history of glucocorticoid withdrawal within weeks or months prior to the hospitalization was present in 13 patients (40.6%), a clinical situation highly suggestive of secondary adrenal failure. All patients survived the acute event and were treated by stress doses of glucocorticoids. Six cases were excluded of the study considered as false positive since under glucocorticoid treatment during the ACTH-stimulation test. These patients had symptoms and/or signs that were potentially associated with adrenal insufficiency but none with adrenal crisis. The prevalence of patients with basal cortisol < 165 nmol/l was of 8.9% (25/281 patients). In the 32 individuals with blunted response to the high-dose ACTH stimulation, the prevalence of low basal cortisol was of 59.4% (19/32 patients). In secondary adrenal insufficiency, the morning cortisol value < 100 nmol/l may indicates adrenal failure. If this criteria was used in our study, the prevalence of secondary adrenal failure diagnosed was of 5.0% (14/281 patients) for the entire cohort and of 43.8% (14/32 patients) for the proven patients with adrenal failure on ACTH-stimulation test.

**Table 1:** Clinical characteristics of the patients with adrenal insufficiency, versus control group

	**All**	**No adrenal insufficiency**	**Adrenal insufficiency**	***P*value**
Number of patients	281	249	32	
Women	132 (47.0)	113 (45.4)	19 (59.4)	0.19
Age (mean)	67 ± 16	68 ± 16	63 ± 15	0.10
Symptoms				
Abdominal pain	35 (12.5)	30 (12.1)	5 (15.6)	0.57
Hypotension symptoms	12 (4.3)	12 (4.8)	0 (0.0)	0.37
Fatigue	48 (17.1)	40 (16.1)	8 (25.0)	0.31
Glucocorticoid withdrawal	53 (18.9)	36 (14.5)	17 (53.1)	<0.001
Nausea	21 (7.5)	16 (6.4)	5 (15.6)	0.07
Generalized weakness	40 (14.2)	33 (13.3)	7 (21.9)	0.19
Signs				
Eosinophilia	2 (0.7)	1 (0.4)	1 (3.1)	0.22
Low blood pressure	141 (50.2)	125 (50.2)	16 (50.0)	0.87
Vitiligo	2 (0.7)	1 (0.4)	1 (3.1)	0.22
Biological markers				
Acidosis	7 (2.5)	7 (2.8)	0 (0.0)	1.00
Hypercalcaemia	10 (3.6)	10 (4.0)	0 (0.0)	0.61
Hyperkaliemia	35 (12.5)	28 (11.2)	7 (21.9)	0.09
Hyponatremia	87 (31.0)	80 (32.1)	7 (21.9)	0.31

Definitions: low blood pressure (<100 mmHg systolic and/or <50 mmHg diastolic blood pressure), vitiligo and/or skin changes. Biological markers collected were: hyperkalemia (>5 mmol/l), hyponatremia (<135 mmol/l), acidosis (pH <7.35), hypercalcaemia (total and ionized, corrected calcium >2.5 mmol/l) and eosinophilia (>0.5 G/l). Results are expressed as number of patients and (percentage). Statistical analysis performed using chi-square or Fischer’s exact test.

On bivariate analysis, history of glucocorticoid withdrawal was the only variable that was significantly different between responders and non-responders to ACTH: OR 6.71, 95% CI [3.08; 14.62]. The duration and the dose of the treatment and the time elapsed between the end of the treatment and the withdrawal was very variable between the patients, and sometimes the information was lacking. None of the abnormal responders were severly stressed, based on C reactive protein, albumin or leucocytosis values.

### Multivariate analysis of factors associated with adrenal insufficiency

The variables significantly and independently associated with adrenal insufficiency were assessed by forward stepwise logistic regression using two significance levels for entry (0.05 and 0.10) (Table 2). In the “restricted” model (RM), only history of glucocorticoid withdrawal (OR 6.66, 95% CI [2.94; 15.09], *p-value* <0.001) and nausea (OR 3.48, 95% CI [1.09; 11.14], *p-value* 0.036) were significantly and independently associated with adrenal insufficiency. In the “broad” model (BM), two more variables were selected: eosinophilia (OR 17.6, 95% CI [1.02; 302.3], *p-value* 0.048) and hyperkalemia (OR 2.41, 95% CI [0.87; 6.69], *p-value* 0.092) (with a history of glucocorticoid withdrawal (OR 7.38, 95% CI [3.18 ; 17.11], *p-value* <0.001) and nausea (OR 3.37, 95% CI [1.03 ; 11.00], *p-value* 0.044)).

**Table 2:** Variables associated with adrenal insufficiency

	**Odds-ratio**	**9%5CI**	***P*value**
Restricted model			
Glucocorticoid withdrawal	6.66	[2.94 ; 15.09]	<0.001
Nausea	3.48	[1.09 ; 11.14]	0.036
Broad model			
Glucocorticoid withdrawal	7.38	[3.18 ; 17.11]	<0.001
Nausea	3.37	[1.03 ; 11.00]	0.044
Eosinophilia	17.6	[1.02 ; 302.3]	0.048
Hyperkaliemia	2.41	[0.87 ; 6.69]	0.092

Results are expressed as Odds ratio and (95% confidence interval) for a yes vs. no variable. Statistical analysis by stepwise forward logistic regression using a significance value <0.05 (restricted model) or <0.10 (broad model) for entry. CI, confidence interval.

The AROCs (95% CIs) were 0.72 (0.66; 0.77); 0.75 (0.70; 0.80) and 0.79 (0.70; 0.89) for the “restricted” model, the “broad” model and the model with all variables, respectively (p = 0.07) (Table 3). Sensitivity and specificity of the RM were 3.1 (0.1 – 16.2) and 99.6 (97.8 – 100), respectively. For the BM, they were 6.3 (0.8 – 20.8) for sensitivity and 99.2 (97.1 – 99.9) for specificity.

**Table 3:** Discrimination capacity of the restrictive and the broad models

	**Restrictive model**	**Broad model**
AROC	0.717 (0.662–0.771)	0.753 (0.700–0.804)
Sensitivity (%)	3.1 (0.1–16.2)	6.3 (0.8–20.8)
Specificity (%)	99.6 (97.8–100)	99.2 (97.1–99.9)
Positive predictive value (%)	50.0 (1.3–98.7)	50.0 (6.8–93.2)
Negative predictive value (%)	88.9 (84.6–92.3)	89.2 (84.9–92.6)
Correctly classified (%)	87.3	88.6

Results are expressed as value and (95% confidence interval). AROC, area under the receiver operating characteristic curve. Restrictive model: glucocorticoid withdrawal and nausea; broad model: glucocorticoid withdrawal, nausea, eosinophilia and hyperkalemia.

In order to assess whether the stepwise model selection was effective, we then performed a model comparison between our models and new models created by adding each time an extra variable (Additional file 1: Table S1). There was not any substantial increase of the AROC, and the differences were not significant, indicating that our model selection was efficient.

## Discussion

The administration of supraphysiologic doses of ACTH (250 μg) is the standard challenge to test the adrenal responsiveness. This test has been widely used and several studies have reported an excellent agreement between peak cortisol concentrations obtained during the test and in the gold standard insulin tolerance test [15,16]. The low-dose (1 μg) ACTH test has been proposed as a more sensitive test to detect secondary adrenal failure. When compared to the usual high dose ACTH test, the 1 μg stimulation test has demonstrated a slightly improved sensitivity. However, the handling of the commercially available ampoule of 250 mg of ACTH makes the test more difficult to perform. Furthermore, it has been suggested that the low dose ACTH may bind to the surface of the injection devices and may blunt the anticipated response [17].

Slightly over one out of ten (11.4%) patients with suspected adrenal insufficient admitted in an acute medical ward had an abnormal response to high-dose ACTH stimulation test defined as a 550 nmol/l cut-off for cortisol. A low baseline cortisol (< 165 nmol/l) not responding to ACTH is sometimes a recognized criteria to establish adrenal insufficiency. Partial secondary adrenal insufficiency might be present in critically ill patients, characterized by a poor cortisol response to ACTH despite normal baseline cortisol. A low cortisol increment (< 248 nmol/l) was measured in 19.2% (54/281 patients) of the entire cohort and in 68.8% (22/32 patients) of the individuals with proven adrenal failure. We cannot rule out that some patients with low cortisol increment to the ACTH challenge may have some functional adrenal insufficiency. However, this was probably not the case in our selected population hospitalized in a general internal medicine ward. All critically ill patients were excluded from the study since these patients are directly admitted in the intensive care unit of our institution. Low blood pressure was present in half of patients in both cases with adrenal insufficiency and controls. This sign was – in the design of our study - not very useful to discriminate between patients with or without adrenal insufficiency. Low blood pressure remains a well recognized typical sign of acute adrenal insufficiency but clinicians should of course rule out other clinical diagnosis responsible for low blood pressure [1,4].

Two multivariate models were analyzed. The “restricted” model was composed of history of glucocorticoid withdrawal and nausea, while the “broad” model included two more variables, eosinophilia and hyperkalemia. Still, the AROCs of both models did not differ, as they did not differ from a model including all signs and symptoms. Although the more parsimonious “restricted” model might be preferred, its screening capacity is not optimal. Hence, in order to maximise the likelihood of correctly diagnosing adrenal insufficiency, clinicians should not rely their judgement on the presence or absence of these two criteria only. The diagnosis of adrenal failure is usually obvious in patients with full-blown symptoms and signs associated with acute glucocorticoid deprivation. However, the diagnosis may be much more difficult in patients admitted in an acute medical ward where multiple co-morbidities are often present. Therefore, the clinician judgement remains a critical step in the evaluation and the “broad model” may therefore be more accurate.

Our study has several limitations. First, it was limited to an acute general internal medicine ward and the estimated prevalence might be different from other wards such as intensive care units. Second, some cases might have been missed if the diagnosis was omitted, namely where the test was not ordered. Finally, the plasma ACTH values were not collected for all individuals and we could not discriminate between primary and secondary adrenal insufficiencies. We also cannot rule out that partial adrenal failure may have been missed in the controls. The high dose ACTH test may identify only profound adrenal failure but not partial and subtle corticotropic failure that could have been detected in a low-dose (1 μg) ACTH stimulation challenge.

## Conclusion

11.4% of suspected adrenal insufficient patients admitted to acute medical ward actually present with adrenal insufficiency, defined by an abnormal response to high-dose (250 μg) ACTH-stimulation test.

Our results suggest that history of glucocorticoid withdrawal is the factor most associated with adrenal insufficiency. The combination of a history of glucocorticoid withdrawal, nausea, eosinophilia and hyperkalemia might be of interest to suspect adrenal insufficiency.

## Abbreviations

ACTH: Adrenocorticotropic hormone; AROC: Area under the Receiver Operating Characteristic curve; OD: Odd ratio; CI: Confidence interval; BM: Broad model; RM: Restricted model.

## Competing interests

The authors declare that they have no competing interests.

## Authors’ contributions

GW and JBO conceived of the study, and participated in its design and coordination and drafted the manuscript. PMV participated in the design of the study, performed the statistical analysis (with JBO) and helped to draft the manuscript. FP participated in the design of the study and helped to draft the manuscript. All authors read and approved the final manuscript.

## Authors’ information

GW is the Professor heading the internal medicine ward of the University Hospital in Lausanne, Switzerland. FP is the Professor heading the endocrinology ward of the University Hospital in Lausanne, Switzerland. PMV is a MD/PhD and has a heading position in the University institute for preventive and social medicine of the University Hospital in Lausanne, Switzerland. JBO is studying in medical school, in the 6^th^ year.

## Pre-publication history

The pre-publication history for this paper can be accessed here:

http://www.biomedcentral.com/1472-6823/13/3/prepub

## Supplementary Material

Additional file 1 Table S1 Effect of including additional variables in the discrimination capacity of the restricted and the broad model. Click here for file
