# Supplementary material for: Predictive factors of adrenal insufficiency in patients admitted to acute medical wards: a case control study
Source: BMC Endocr Disord. 2013 Jan 26;13:3. doi: 10.1186/1472-6823-13-3 (PMC3574842; doi:10.1186/1472-6823-13-3)
Supplement: Additional file 1 Table S1 — Effect of including additional variables in the discrimination capacity of the restricted and the broad model. [file 1472-6823-13-3-S1.doc]

**Additional Table S1**: Effect of including additional variables in the discrimination capacity of the restricted and the broad model.

| **Variable added** | **Restrictive model** | **Broad model** |
| --- | --- | --- |
| Model alone | 0.717 (0.662 – 0.771) | 0.753 (0.700 – 0.804) |
| + age | 0.743 (0.645 – 0.841) | 0.782 (0.693 – 0.871) |
| + female | 0.729 (0.620 – 0.837) | 0.759 (0.657 – 0.861) |
| symptoms |  |  |
| + abdominal pain | 0.726 (0.631 – 0.820) | 0.751 (0.654 – 0.847) |
| + hypotension symptoms | NA | NA |
| + fatigue | 0.736 (0.642 – 0.830) | 0.763 (0.668 – 0.857) |
| + altered general well being | 0.737 (0.644 – 0.830) | 0.767 (0.678 – 0.857) |
| signs |  |  |
| + eosinophilia | 0.733 (0.643 – 0.823) | - |
| + low blood pressure | 0.707 (0.598 – 0.816) | 0.747 (0.642 – 0.852) |
| + vitiligo | 0.718 (0.626 – 0.811) | 0.754 (0.660 – 0.848) |
| biological markers |  |  |
| + acidosis | NA | NA |
| + hypercalcaemia | NA | NA |
| + hyperkalaemia | 0.736 (0.641 – 0.832) | - |
| + hyponatraemia | 0.733 (0.639 – 0.827) | 0.760 (0.667 – 0.853) |

Results are expressed as area under the receiver operating characteristic curve and (95% confidence interval). NA, not possible to be assessed; -, already included in the model.
